# Fear and Practice Modifications among Dentists to Combat Novel Coronavirus Disease (COVID-19) Outbreak

**DOI:** 10.3390/ijerph17082821

**Published:** 2020-04-19

**Authors:** Muhammad Adeel Ahmed, Rizwan Jouhar, Naseer Ahmed, Samira Adnan, Marziya Aftab, Muhammad Sohail Zafar, Zohaib Khurshid

**Affiliations:** 1 Department of Restorative Dentistry and Endodontics, College of Dentistry, King Faisal University, Al-Ahsa 31982, Saudi Arabia; rjouhar@kfu.edu.sa; 2 Department of Prosthodontics, Altamash Institute of Dental Medicine, Karachi 75500, Pakistan; naprosthodontist@gmail.com; 3 Department of Operative Dentistry, Sindh Institute of Oral Health Sciences, Jinnah Sindh Medical University, Karachi 75510, Pakistan; samira.adnan@jsmu.edu.pk; 4 Department of Operative Dentistry, Dr. Ishrat-ul-ebad khan Institute of Oral Health Science, Dow University of Health Sciences, Karachi 74200, Pakistan; marziya.malik@gmail.com; 5 Department of Restorative Dentistry, College of Dentistry, Taibah University, Al-Madina Al-Munawwarah 41311, Saudi Arabia; MZAFAR@taibahu.edu.sa; 6 Department of Dental Materials, Islamic International Dental College, Riphah International University, Islamabad 44000, Pakistan; 7 Department of Prosthodontics and Dental Implantology, College of Dentistry, King Faisal University, Al-Ahsa 31982, Saudi Arabia; drzohaibkhurshid@gmail.com

**Keywords:** fear, dental practice, coronavirus

## Abstract

An outbreak of novel coronavirus disease (COVID-19) in China has influenced every aspect of life. Healthcare professionals, especially dentists, are exposed to a higher risk of getting infected due to close contact with infected patients. The current study was conducted to assess anxiety and fear of getting infected among dentists while working during the current novel coronavirus diseases (COVID-19) outbreak. In addition, dentists’ knowledge about various practice modifications to combat COVID-19 has been evaluated. A cross-sectional study was conducted using an online survey from 10th to 17th March 2020. The well-constructed questionnaire was designed and registered at online website (Kwiksurveys) and validated. A total of 669 participants from 30 different countries across the world responded. After scrutiny, completed questionnaires (n = 650) were included in the study. Statistical analysis was performed using SPSS version 25. Chi-Square and Spearman correlation tests were applied to control confounders and assess the relation of dentists’ response with respect to gender and educational level. More than two-thirds of the general dental practitioners (78%) from 30 countries questioned were anxious and scared by the devastating effects of COVID-19. A large number of dentists (90%) were aware of recent changes in the treatment protocols. However, execution of amended treatment protocol was recorded as 61%. The majority of the dentists (76%) were working in the hospital setting out of which 74% were from private, and 20% were from government setups. Individually we received a large number of responses from Pakistan and Saudi Arabia, but collectively more than 50% of the responses were from other parts of the world. Despite having a high standard of knowledge and practice, dental practitioners around the globe are in a state of anxiety and fear while working in their respective fields due to the COVID-19 pandemic impact on humanity. A number of dental practices have either modified their services according to the recommended guidelines to emergency treatment only or closed down practices for an uncertain period.

## 1. Introduction

An outbreak of novel coronavirus disease (COVID-19) in China has influenced every aspect of life [1]. Within a few months, COVID-19 has spread globally and on 11th March 2020, the World Health Organization (WHO) declared it as a controllable pandemic disease [2,3]. The latest strain of coronavirus is believed to have originated in a seafood market in Wuhan, China [1]. On 11th February 2020, WHO used the term COVID-19 to describe the latest strain of coronavirus [4].

Structurally, COVID-19 is an ss-RNA, enveloped virus with a size of ~350 kilobase-pair (kbp) [5]. COVID-19 has the potential to cause severe acute respiratory tract infection among infected humans and is commonly transmitted from person to person via hands, saliva, nasal droplets, and surface contacts [3,6]. The average incubation period ranges of COVID-19 from 4 to 14 days [7]. The infected person usually presents with upper respiratory tract infection (RTI) and complaints of high-grade fever, a dry cough, and dyspnea [8]. It is highly recommended to keep any suspected individuals in quarantine (isolation) and under observation until further investigation by the real-time polymerase chain reaction (RT-PCR) can take place [9]. Unfortunately, there is no antiviral vaccine available on the market, but on 16th March 2020 the first clinical trial was initiated by the National Health Institute (NHI), USA [3]. Therefore, patients have to rely on supportive therapy such as vitamins A, C and D; chloroquine phosphate; and general healthcare until the body’s immune system can eradicate the infection [10].

Considering the vital role of the body’s immune system, elderly patients with chronic debilitating diseases have a higher risk of getting infected compared to young, healthy individuals with a strong immune system [11]. To date, three quarter of a million cases have been reported, and more than thirty-three thousand patients have died around the world (Source WHO situation report-70). Although the mortality associated with COVID-19 is low, it has a high spreading potential [12]. Since the COVID-19 outbreak is so fast and devastating, many countries have shut down teaching institutions, social gatherings, sports activities, events, airports, and even banks in an attempt to control the spread of the infection. Besides this, several individuals went into self-quarantine in order to play their part in society by limiting the spread of disease. 

On the other hand, healthcare facilities are necessarily required for any society and are rarely closed under such pandemic conditions. Healthcare professionals are exposed to a higher risk of getting infected due to their close contact with infected patients [13]. In particular, dentists perform their duties not only in close contact with patients but also while exposed to aerosol and droplets splashing out of patients’ oral cavity [13,14]. Therefore, dentists have a high risk of getting infected from patients and potentially spreading it to their peers, families, and other patients. Under these circumstances, it may be natural for dentists to develop a fear of being infected by their patients. 

Fear and anxiety are powerful emotions that may be associated with the overwhelming reports on the COVID-19 pandemic by social, electronic, and print media. Mild anxiety is natural and fosters preventive and safeguarding behavior [15]. At the current juncture, people with persistent anxiety may panic and are more likely to make mistakes leading to irrational decisions and behavior. Being on the list of high-risk professions, dentists are very much expected to develop severe anxiety about the current pandemic situation [16]. Considering the current rapid spread of infection, the American Dental Association (ADA) highlighted key steps to be taken by dentists in addition to the standard universal precautions such as taking patients’ recent travel history; assessing signs and symptoms of RTI; recording patients’ body temperature; mouth rinsing with 1% hydrogen peroxide prior to commencement of any procedure; using a rubber dam and high volume suction during procedures; and frequently cleaning and disinfecting public contact areas including door handles, chairs and, washrooms [13]. Although the ADA has published preventive guidelines, the majority of dentists are still reluctant and feel fearful of treating patients in such a situation (Source ADA-COVID-19 Resources for Dentists). In fact, most dentists may not be aware of the recent guidelines. Therefore, we have conducted a questionnaire-based study to evaluate dentist response globally. The present study aimed to assess anxiety and fear of getting infected among dentists working during the current viral outbreak. In addition, dentist knowledge about various practice modifications to combat the novel coronavirus disease (COVID-19) outbreak has been evaluated.

## 2. Materials and Methods 

**Figure 1:**
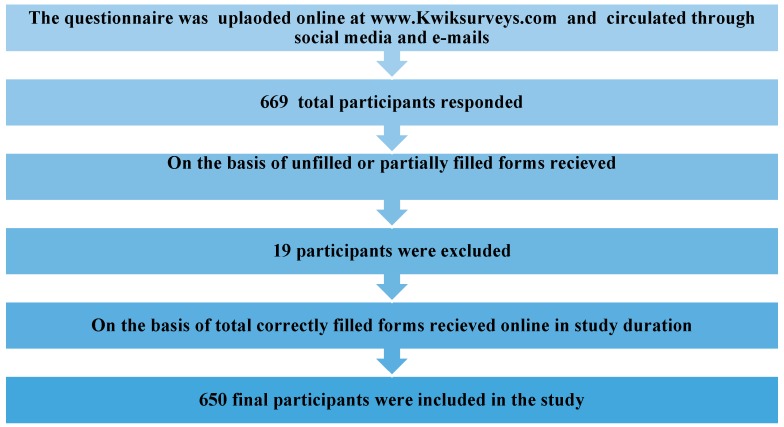
Flow chart of participant’s recruitment.

The present cross-sectional study was conducted using an online survey questionnaire from 10th to 17th March 2020. For this purpose, a well-constructed questionnaire was designed at www.Kwiksurveys.com and validated through intra-class correlation with a strong relation of 0.74. The online survey link was circulated through social media and an e-mail to dental professionals and received a response through an online survey submission. Any paramedical staff and dental students were not included in this survey. The questionnaire was comprised of a total of 22 closed-ended questions, which were divided into two sections. The first section focused on the fear among dentists about getting infected with COVID-19 and the second section was designed to gather information about their practice modifications to combat COVID-19 outbreak in accordance with the Centers for Disease Control and Prevention (CDC) and ADA practice guidelines. A total of 669 participants from 30 different countries across the world participated and submitted the questionnaire, excluding 19 unfilled or partially filled forms (Figure 1). An ethical review board approved the study (AIDM/EC/03/2020/20) and statistical analysis was done on SPSS version 25. A Chi-Square and Spearman Correlation test were used to control confounders and assess the relation of dentist response with respect to concerning gender and education level.

## 3. Results

**Figure 2:**
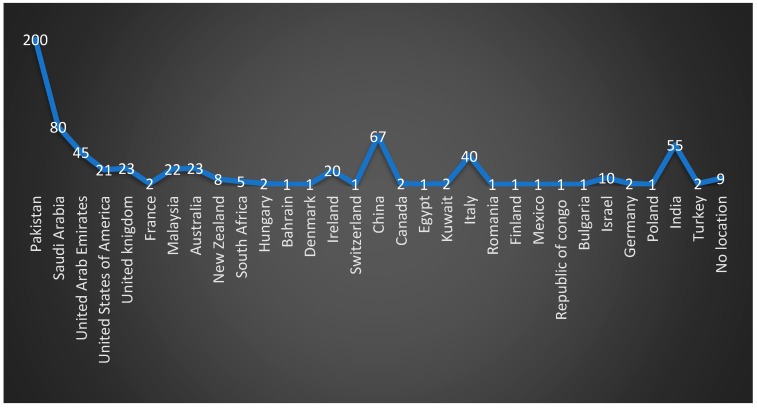
Distribution of participants’ responses by county (n = 650).

A total of 650 participants from 30 countries worldwide submitted the completed questionnaire with a total of 22 questions comprising of two sections about fear or anxiety level and practice modification due to COVID-19. The responses were recorded from various countries as follows: Saudi Arabia (80); Pakistan (200); India (55); United Arab Emirates (45); People’s Republic of China (67); Italy (40); United Kingdom (23); Australia (23); Malaysia (22); United States of America (21); Ireland (20); Israel (10); New Zealand (08); South Africa (05); Turkey, Germany, Kuwait, Canada, Hungary, and France recorded (02) each; and Poland, Bulgaria, Republic of the Congo, Mexico, Finland, Romania, Egypt, Switzerland, Demark, and Bahrain recorded (01) each. There were (09) responses from unidentified locations (Figure 2).

The demographic information of the participants is presented in Table 1. Out of a total of 650 participants, 160 were male and 490 female, with a common age bracket between 20 and 40 years (92.84%). By designation, 511 were general dentists, 97 specialists, and only 42 were from the consultant category. Similarly, 511 were graduates and 139 postgraduates by qualification while 482 dentists were from a private setup, 37 were semiprivate and 131 from a government work setting. The majority of participants (495) were working in a hospital while 155 were working in clinics (Table 1). The present study reported no significant relationship (rho-0.2), (P 0.06) between dental care professionals’ responses with gender and their education level.

In Table 2 there is a description of the fear and anxiety levels of dental care professionals towards COVID-19; 87% of participants were afraid of getting infected with COVID-19 from either a patient or a co-worker. While treating a coughing or a patient suspected to be infected with COVID-19, 90% were anxious. More than 72% of participants felt nervous when talking to patients in close vicinity, 92% were afraid of carrying the infection from dental practice to their families, and 77% were afraid of getting quarantined if they got infected. The anxiety rate concerning the cost of treatment if they got infected was 73%, while 86% felt afraid while they learnt about mortalities because of COVID-19. A considerable number of dentists (66%) wanted to close their dental practices until the number of COVID-19 cases start to decline.

In Table 3 there is a description of the knowledge of dental care professionals about COVID-19; 97% were aware of its mode of transmission, and 90% were updated with the current CDC or WHO guidelines for cross-infection control. Accordingly, 82% preferred asking about the patient’s travel history, 81% recorded every patient’s body temperature before performing dental treatments, and 78% deferred dental treatment of patients who disclosed suspicious symptoms. In terms of using personal protection, 85% believed that a surgical mask is not enough to prevent cross-infection of COVID-19. In comparison, 84% favored the use of N-95 masks for routine dental procedures during the current outbreak. On the contrary, 90% reported not wearing an N-95 mask while treating a patient. Although the majority (89%) recommended routine universal precautions of infection control, 84% did not use rubber dam isolation for every patient. Seventy-six percent of participants used high-volume suction for every patient. However, 74% did not ask patients to rinse the mouth with antibacterial mouthwash before dental treatment. Ninety-four percent of participants practiced washing hands with soap and water or sanitizer before and after treatment of patients, while 80% of participants were aware of the proper authority to contact if they came across a patient with a suspected COVID-19 infection.

## 4. Discussion

The present cross-sectional study reported the anxiety and fear of getting infected among dentists while working during the current viral outbreak. For this purpose, a questionnaire focusing on closed-ended questions was used to gather information about dentist’s fear and any practice modifications to combat the COVID-19 outbreak epidemic. Questionnaire-based studies are proven for gathering information regarding preferences, attitudes, opinions, and experiences of participants; however, careful data collection and interpretation is required [17]. The questionnaire used in the present study collected information objectively and validated through intra-class correlation with a strong relation of 0.74. Any incomplete questionnaires were excluded. Psychological implications such as fear and anxiety are natural in pandemics, especially when the number of infected individual and mortality rates are increasing sharply. Studies on previous outbreaks of similar infectious diseases such as severe acute respiratory syndrome (SARS) demonstrated various factors leading to psychological trauma in healthcare workers including the fear of getting infected while treating an infected patient, or infecting a family member [18,19]. The repercussions of the current rapid spread of COVID-19, which has affected millions of people worldwide, ranging from being isolated and quarantined to fatality has resulted in considerable psychological stress and fear. With the prolonged incubation period of the coronavirus (as long as 14 days), it is virtually impossible to pinpoint an individual’s exposure to the virus [20]. In addition, there is no vaccine or approved treatment, which further enhances anxiety upon the thought of getting infected. Healthcare workers dealing with sick patients continuously are at a higher risk of acquiring infectious diseases, adding a tremendous psychological toll [21].

Since it has been established that the primary route for transmission of coronavirus is through droplets and aerosols [22], this enhances the likelihood of dentists and dental healthcare workers of getting infected and further spreading the virus. The current study found that a large number of dentists fear getting infected by their patients or co-workers. The response is similar to the perception of rest of the population where people are terrified of getting infected from other individuals in the community in the presence of a rapidly developing epidemic [23]. The majority of the dentists are fearful of providing treatment to any patient reporting suspicious symptoms. Since COVID-19 has rapidly infected such a large number of individuals in almost every country, the fear of getting infected by a patient is justified. The high level of anxiety was reflected as a large proportion of dentists wanted to close down their practices which may have significant economic implications for dentists and dental healthcare workers. In addition, patients suffering from dental pain and/or undergoing a multi-visit treatment plan may have to experience delays in dental care in such circumstances. The current guidelines on the COVID-19 outbreak have recommended deferring all non-essential dental treatment, and only patients suffering from pain, swelling, bleeding, and trauma are advised to undergo treatment [14,24]. In the current scenario, all elective or non-essential dental treatment for all patients should be deferred until the situation is regressing or under control [14]. There is a study conducted at a dental emergency department in Beijing, China [25], where they have observed an impact of the COVID-19 pandemic on the reporting of dental treatments, which has declined in the emergency department as compared to reporting pre-COVID-19. Due to the COVID-19 pandemic, less dental trauma has been reported and the proportion of dental and oral infection has increased while those of dental trauma and non-urgency have decreased [25]. 

Another genuine fear that dentists have is of carrying infections from their dental practices to their families. The coronavirus can last on various surfaces for a few hours to a few days [26]. This, combined with its prolonged incubation period before symptoms develop, are factors that make it particularly difficult to limit its transmission. The trepidation of getting quarantined as a result of suspected disease or actual infection is also a legitimate fear when one thinks about how the rest of the family members are likely to suffer due to various aspects. The burden on the healthcare system and cost incurred during treatment also puts one’s mind at stress. Health facilities may not be state-sponsored globally and hence can result in a significant financial burden. The screening and testing for COVID-19 have been greatly subsidized by the government bodies in many countries worldwide, which is encouraging residents to get tested for COVID-19 in cases of suspected infection. 

A positive aspect reposted by the present study was that the majority of the participants were aware of the COVID-19 mode of spread and transmission. As a part of infection control measures, such information is essential during dental practice. It is crucial in the face of this pandemic to further follow the procedures and guidelines focusing on reducing the amount of aerosol generated and deal with it effectively. Similarly, it was encouraging that a large number of dentists were aware of the current guidelines issued by the Center for Disease Control (CDC) and WHO for cross-infection control in the dental practice including asking patients’ travel history and recording patients’ body temperature [8]. Understandably, both these facts may provide a fair idea of potentially infected patients and their precautionary management in dental practice. Of course, the routine universal precautions already recommended and endorsed by various regulatory and infectious control authorities worldwide for the prevention of cross-infection in dental practice should be rigorously adhered to in the current circumstances. Although the majority of dentists agreed that these precautions should be practiced for every patient, unfortunately, a large number of participants reported not using basic cross-infection measures like the rubber dam for every patient. Use of a rubber dam is an effective way to control cross-infection by limiting the spread of aerosols with good patient acceptance for dental procedures [24]. Considering the benefits, there is no excuse for not using rubber dam during dental procedures, especially while using rotary instruments that generate a large quantity of aerosols and droplets. The use of high-volume suction is also considered an essential means to control aerosols evacuation during dental procedures and should be used for the majority of patients [22].

At the start of any dental procedure, rinsing with an antimicrobial mouthwash also significantly reduces the microbial load [27,28]. This practice is recommended in the current pandemic, however the majority of dentists reported ignoring it. At present there is no available evidence addressing the effects of commonly used antimicrobial mouth rinses on COVID 19. Hence, this recommendation could be based on the fact that gargling has been reported to decrease the viral load and spread by removing oropharyngeal protease and associated viral replication [29]. In addition, mouthwashes containing agents with anti-viral activity such as povidone-iodine have exhibited effectiveness against various respiratory viruses [30,31].

During the outbreak of COVID-19, the importance of hand hygiene has been emphasized repeatedly and this is even more important in the case of dental practitioners. Studies have shown that proper hand hygiene, including handwashing with soap and water and cleaning using alcohol-based sanitizers, is an essential measure in controlling the spread of respiratory illness including SARS [32,33]. Therefore, WHO recommends frequent hand washing or using an alcohol-based hand sanitizer in the dental practice. The use of a particulate respirator such as the N-95 mask has been recommended for treating patients suspected of COVID-19. Otherwise, at least a surgical mask must be used while treating all patients when the distance between the dental healthcare worker and the patient is less than 1 m [22].

Currently, recommendations are based on experiences and pertinent guidelines in addition to universal precautions applied to all dental patients. Additionally, the intra-oral radiographs may be replaced by extra-oral radiographs such as orthopantomogram, and cone beam computed tomography where possible. Periodontal procedures utilizing ultrasonic scalers should be substituted with hand scalers aiming to reduce the production and spread of aerosol and splatter. Besides the regular use of a rubber dam, high-volume suction helps to keep aerosols in check and prevent droplets originating in the patient’s oral cavity and respiratory tract from spreading and potentially transmitting infection. Currently, around the world, dental regulatory authorities such as the ADA are urging dentists to conduct only emergency dental treatments. Further recommendations for dental treatment can be found elsewhere in relevant documents [6].

It is crucial in the face of the fear and anxiety shown by the dental community towards COVID-19 that psychological coping mechanisms and strategies are practiced in order to remain calm and function efficiently. The fear that dentists have regarding getting infected from COVID 19 could be greatly curtailed if dentists and dental healthcare workers meticulously follow the relevant recommendations issued by the regulatory authorities. These include the universal cross-infection control protocols along with some additional precautions in cases where patients present with any suspicious symptoms.

## 5. Limitations

Some of the limitations of this study is data was collected in a concise duration of time, keeping in mind the rapid effect this outbreak was having on the psychology and practices of dental health practitioners. It may be argued that the attitudes and knowledge of dentists may alter with the emerging research and possible treatment of COVID-19. Furthermore, we did not receive responses from all countries that have been affected by the outbreak. Hence, the generalizability of the study is limited. Although the present study included participants from various countries across the globe, every country may have variable information, policies, and guidelines regarding COVID-19 that may directly influence participant responses. Similarly, some countries are more affected than others which may affect administrative, precautionary, and healthcare measures taken by a specific country that can also influence the outcome of a survey. Therefore, the findings of the present study should be interpreted carefully and not be globalized. Even though the questionnaire was sent to dentists almost all over the world, there was a lack of response mainly from European and African countries and resultant small sample size. The reason for this could be that the current panic may have diverted the attention of potential respondents towards other priorities, possibly related to the continuing or imminent lockdown conditions in many countries. Due to the cross-sectional nature of the study plan, we couldn’t conclude a cause–effect relationship.

## 6. Conclusions

Despite having high standards of knowledge and practices, dental practitioners around the globe are in a state of anxiety and fear while working in their respective fields due to the COVID-19 pandemic impact on humanity. Currently, the effects of COVID-19 around the globe are worsening day by day. Several dental practices have either modified their services according to recommended guidelines to emergency treatment only, or closed down practices for an uncertain period. It is essential that in the present scenario, priority is given to dental procedures labeled as emergencies by the WHO and that all dental treatments are deferred until a time when the outbreak goes into recession. This would be an appropriate step in attempts to curtail the further spread of COVID-19.

## Figures and Tables

**Table 1:** Demographic information of dental care professionals n = 650.

Demographics		Number (%)
**Gender**	Male	160 (25)
	Female	490 (75)
**Age**	20 to 30	350 (54)
	31 to 40	254 (39)
	41 to 50	37 (5)
	51 to 60	8 (1.85)
	Above 60	1 (0.15)
**Designation**	General Dental Practitioner	511 (78)
	Specialist	97 (14)
	Consultant	42 (8)
**Qualification**	Graduates	511 (78)
	Postgraduates	139 (22)
**Workplace**	Hospital	495 (76)
	Clinic	155 (24)
**Work Setting**	Private	482 (74)
	Semiprivate	37 (6)
	Government	131 (20)

**Table 2:** Fear and anxiety assessment of dental care professionals n= 650.

	Yes n (%)	No n (%)	Unaware n (%)
**Are You Afraid of Getting Infected with COVID-19 from a Patient and Co-Worker?**	566 (87)	66 (10)	18 (3)
**Are You Anxious When Providing Treatment to a Patient who is Coughing or Suspected of being Infected with COVID-19?**	585 (90)	48 (7)	17 (3)
**Do You want to Close Your Dental Practice until the Number of COVID-19 Cases Starts Declining?**	431 (66)	153 (23)	66 (11)
**Do You Feel Nervous when Talking to Patients in Close Vicinity?**	467 (72)	160 (24)	23 (4)
**Do You have Fear that You Could Carry the Infection from Your Dental Practice back to Your Family?**	600 (92)	40 (6)	10 (2)
**Are You Afraid of Getting Quarantined if get Infected**	505 (77)	121 (19)	24 (4)
**Are You Anxious about the Cost of Treatment if You Get Infected?**	476 (73)	141 (22)	33 (5)
**Do You feel Afraid when you Hear that People are Dying Because of COVID-19?**	547 (85)	87 (12)	16 (3)

**Table 3:** Knowledge and practice of dentists about COVID 19 n = 650.

	Yes n (%)	No n (%)	Don’t Know n (%)
**Are You Aware of the Mode of Transmission of COVID-19?**	631 (97)	15 (2)	4 (1)
**Are You Updated with the Current CDC or WHO Guidelines for Cross-Infection Control regarding COVID-19?**	588 (90)	54 (8)	8 (2)
**Are You currently Asking every Patient’s Travel History before Performing Dental Treatment?**	532 (82)	108 (16)	10 (2)
**Are You currently Taking every Patient’s Body Temperature before Performing Dental Treatment?**	530 (81)	115 (17)	5 (2)
**Are You Deferring Dental Treatment of Patients Showing Suspicious Symptoms?**	512 (78)	111 (17)	27 (5)
**Do You Think Surgical Mask is enough to Prevent Cross-Infection of COVID 19**	72 (11)	553 (85)	25 (4)
**Do You Think N-90 Mask should be Routinely Worn in Dental Practice due to the Current Outbreak?**	548 (84)	75 (11)	27 (5)
**Have You Ever Worn an N-90 Mask while Treating a Patient in Your Dental Practice?**	58 (9)	586 (90)	6 (1)
**Do You Routinely Follow Universal Precautions of Infection Control for Every Patient?**	583 (89)	58 (9)	9 (2)
**Do You Use Rubber Dam Isolation for Every Patient?**	93 (14)	550 (84)	7 (2)
**Do You Use High-Volume Suction in Your Practice for Every Patient?**	499 (76)	143 (22)	8 (2)
**Do You Ask Every Patient to Rinse His/Her Mouth with Anti-Bacterial Mouthwash before Treatment?**	160 (24)	484 (74)	6 (2)
**Do You Wash Hands with Soap and Water/Use Sanitizer Before and After Treatment of Every Patient?**	611 (94)	37 (5)	2 (1)
**Are You Aware of which Authority to Contact if You Come Across a Patient with Suspected COVID-19 Infection?**	519 (80)	114 (17)	17 (3)
